# Role of ^18^ FDG PET/CT in Detecting Primary Tumors in Patients with Carcinoma of Unknown Primary: Single-Center Cross-Sectional Study from 2017 to 2023 (Extension Study)

**DOI:** 10.1055/s-0044-1795101

**Published:** 2024-11-19

**Authors:** Nosheen Fatima, Mina Laiq, Muhammad Rafay, Sara Muhammad Azam, Maseeh uz Zaman

**Affiliations:** 1 Section of PET/CT Imaging Services, Department of Radiology, Aga Khan University Hospital, Karachi, Pakistan

**Keywords:** carcinoma of unknown primary, CUP, ^18^ FDG PET/CT, detection efficiency, extension study

## Abstract

**Background** Carcinoma of unknown primary (CUP) is a diverse group of cancers in which the primary tumor site remains occult despite detailed investigations. This is an extension of a published parent study with a smaller cohort, to further validate the published facts of detection efficiency of 18F-fluorodeoxyglucose positron emission tomography/computed tomography ( ^18^ FDG PET/CT) in patients with CUP over a larger sample from 2017 to 2023.

**Methods** Patients with CUP referred for ^18^ FDG PET/CT scan for detection of primary sites during the study period were recruited. ^18^ FDG PET/CT scan was acquired using a standardized protocol, and patients with suspected primary sites underwent biopsies. Scan findings and biopsy results were analyzed to find the detection rate, sensitivity, area under the curve (AUC), and positive predictive value (PPV). As no biopsy was performed in cases with negative scan, these cases were considered false negatives (FNs).

**Results** Total 230 patients with CUP were included with similar demographic trend (mean age: 58 ± 14 years; 63% male and 37% female; mean body mass index: 26.82 ± 5.4 kg/m ^2^ ); 138/230 (60 vs. 74% in parent study) patients were found to have a hypermetabolic focus suggestive of primary tumor sites and subjected to biopsy which turned out positive in 127/138 (true positive [TP]: 92 vs. 76% in parent study) and negative in 11/138 (true negative [TN]: 8 vs. 24% in parent study). Sensitivity and PPV of ^18^ FDG PET/CT were 58 and 92%, respectively (68 and 76%, respectively, in parent study). The remaining 92/230 (40%) patients with negative ^18^ FDG PET/CT for primary focus did not have biopsy. No significant demographic difference was seen in patients with TP and FN studies ( *p* > 0.05). Receiver operating characteristics (ROC) curve revealed fair diagnostic strength of ^18^ FDG PET/CT for detecting unknown primary (AUC 0.710; *p* ≤ 0.05; standard error = 0.0167; confidence interval: 0.647–0.768; vs. nonsignificant in parent study).

**Conclusion** We conclude that this extension study with a larger cohort compared with the parent study has found a similar detection efficiency of ^18^ FDG PET/CT for identifying primary tumor in patients with CUP (58 vs. 57%) but with better PPV and sensitivity. Upfront use of ^18^ FDG PET/CT in CUP could preclude the use of many futile diagnostic procedures. Furthermore, the use of tumor-specific PET tracers, higher resolution scanners, and acquiring delayed images in patients with negative ^18^ FDG study could reduce FN results in patients with CUP.

## Introduction

Carcinoma of unknown primary (CUP syndrome) is a diverse group of cancers in which the primary tumor site remains occult despite detailed investigations, and it accounts for 2 to 5% of cancers worldwide. 1 The median age at presentation is 60 to 65 years with a male preponderance by a ratio of 3:2. 2 CUP is usually aggressive in nature with an observed survival of > 1 year in ∼50% of patients. 3 Furthermore, due to extensive investigations, the time from presentation to the start of treatment is longer which results in higher pretreatment costs in patients with CUP. 4 Computed tomography (CT) and conventional magnetic resonance imaging (MRI) are the most common cross-sectional morphological imaging modalities with a detection rate of the primary tumor in 22 to 36% of patients with CUP. 5 This lower detection efficiency is due to smaller and nonenhancing lesions in normal-sized structures that may be missed on conventional CT and MRI. In contrast to conventional imaging modalities, F18-fluorodeoxyglucose positron emission tomography/CT ( ^18^ FDG PET/CT) does not have the above-mentioned limitation as it exploits the enhanced glucose metabolism in many malignant tumors (Warburg effect) to highlight a hypermetabolic focus at occult primary tumor site. 6 Although the relative nonspecificity of ^18^ FDG may pose a challenge, anatomical details provided by its CT component have greatly enhanced the assessment of hypermetabolic foci. 7 Published studies have also shown that in CUP, ^18^ FDG PET/CT has a higher detection rate of primary tumor (24–40%) compared with CT or MRI (20–27%). 8 In a prior study by our group published in 2019 with a smaller cohort (46 patients), we reported a detection rate, sensitivity, and positive predictive value (PPV) of 57, 68,, and 76%, respectively. 9 Now, we are presenting our findings as an extension of the previous study with data collected from August 2017 to December 2023. The purpose of this extension study was to report the detection efficiency of ^18^ FDG PET/CT in a larger cohort of patients with CUP.

## Methods

This is a continuation of a single-center cross-sectional prospective study conducted at the Section of PET/CT Imaging Services, Department of Radiology, Aga Khan University Hospital, Karachi, Pakistan, from August 2017 to January 2024. We included consecutive patients with CUP referred for ^18^ FDG PET/CT scan for detection of primary sites after obtaining informed consent as per institutional ethical review committee policy (2024-9755-28071). All patients had biopsy-proven metastatic disease in whom detailed physical examination, laboratory investigation, CT, MRI, and endoscopic procedures failed to identify the primary tumor. Patients with indeterminate biopsy for malignancy were excluded from the study. Patients with a suspected primary tumor site identified on ^18^ FDG PET/CT underwent biopsy. Scan findings and biopsy results were analyzed to find the detection rate, sensitivity, area under the curve (AUC), and PPV. As no biopsy was performed in a negative scan, TN and specificity could not be calculated.

### ^18^ FDG PET/CT Imaging

^18^ FDG PET/CT was performed as per institutional protocol adopted from European Association of Nuclear Medicine (EANM) guidelines. 10 All patients had at least 6-hour fasting (only plain water was allowed) and a fasting blood sugar (FBS) less than 200 mg/dL before receiving an intravenous ^18^ FDG dose of 3 MBq/kg in the uptake room. During the uptake period (mean 55–75 minutes), patients were requested to lie down comfortably and allowed to take ∼500 to 1,000 mL of plain water. The urinary bladder was emptied prior to calling the patient into the PET/CT imaging suite equipped with a Celesteion, Toshiba, Japan. A low-dose CT examination (mid-brain to mid-thigh) was performed without intravenous contrast from head to toe followed by the acquisition of PET imaging which took 3 minutes/bed position from toe to head in all patients. Both PET and CT images were acquired with patients under normal tidal breathing. PET (both nonattenuation corrected and attenuation corrected), CT, and fusion ^18^ FDG PET/CT images were examined in axial, coronal, and sagittal planes on the manufacturer's review station. All ^18^ FDG PET/CT images were evaluated by two nuclear physicians having an experience of more than 5 years. On a transaxial, attenuation-corrected PET image, the maximum standardized uptake values (SUVmax) were obtained by placing regions of interest on hypermetabolic lesions that had been identified on visual analysis.

### Biopsy of Suspected Primary Tumor

Patients with a hypermetabolic suspected primary tumor site on PET/CT underwent a CT or ultrasound-guided core biopsies. ^18^ FDG PET/CT findings and biopsy results were analyzed to find the detection rate, sensitivity, AUC, and PPV. Biopsy was not considered in patients who had negative ^18^ FDG PET/CT scan for suspected primary tumor.

### Statistical Analysis

Continuous variables were described by mean ± standard deviation. A contingency table was drawn to calculate frequency distribution of true positive (TP), false positive, and false negative (FN). Detection rate, sensitivity, and PPV were calculated. Receiver operating characteristics (ROC) curve was analyzed for diagnostic strength of PET/CT in suggestive primary neoplasm. Statistical significance was defined as *p* < 0.05. Commercially available packages Medcalc 2024 version 22.019 and the Statistical Package for Social Sciences (SPSS 19 Armonk, New York, United States) were used.

## Results

**Fig. 1:**
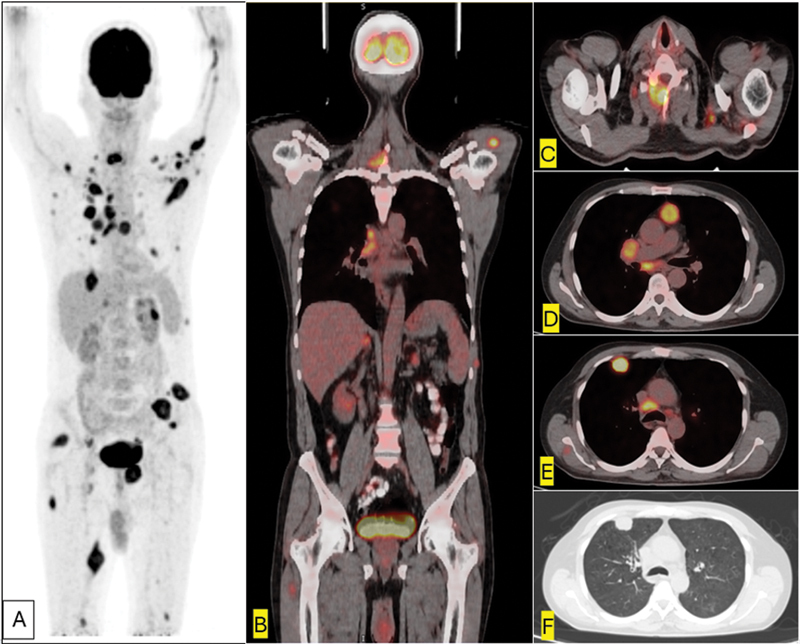
^18^ FDG PET/CT in a male with CUP on C7 biopsy. Maximum intensity projection (A) shows widespread hypermetabolic soft tissue and bony deposits. Fused coronal (B) image shows hypermetabolic lesions over C7, right hilum, and both adrenal (larger on left). Fused axial slices show a destructive lesion involving the right lamina of C7 (C), multiple hypermetabolic metastatic thoracic nodes (D), pleural-based hypermetabolic soft tissue mass in the right upper lobe anteriorly (E, F) which turned out to be small cell lung cancer on subsequent biopsy. CT, computed tomography; CUP, carcinoma of unknown primary; ^18^ FDG, 18F-fluorodeoxyglucose; PET. positron emission tomography.

**Fig. 2:**
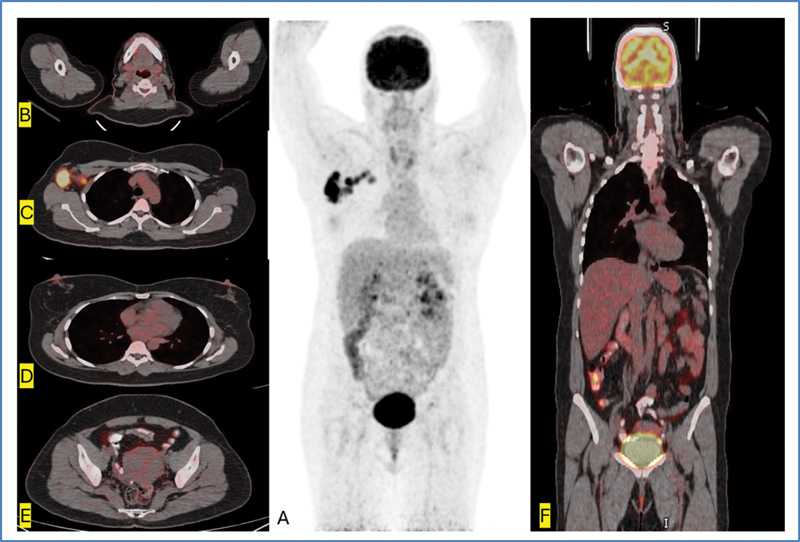
^18^ FDG PET/CT maximum intensity projection (A) and axial fused (C) showing multiple hypermetabolic right axillary nodes (biopsy-proven CUP). Axial fused slices of neck (B), breast (D), pelvis (E), and fused coronal (F) show no hypermetabolic focus to suggest primary tumor. CT, computed tomography; CUP, carcinoma of unknown primary; ^18^ FDG, 18F-fluorodeoxyglucose; PET. positron emission tomography.

**Fig. 3:**
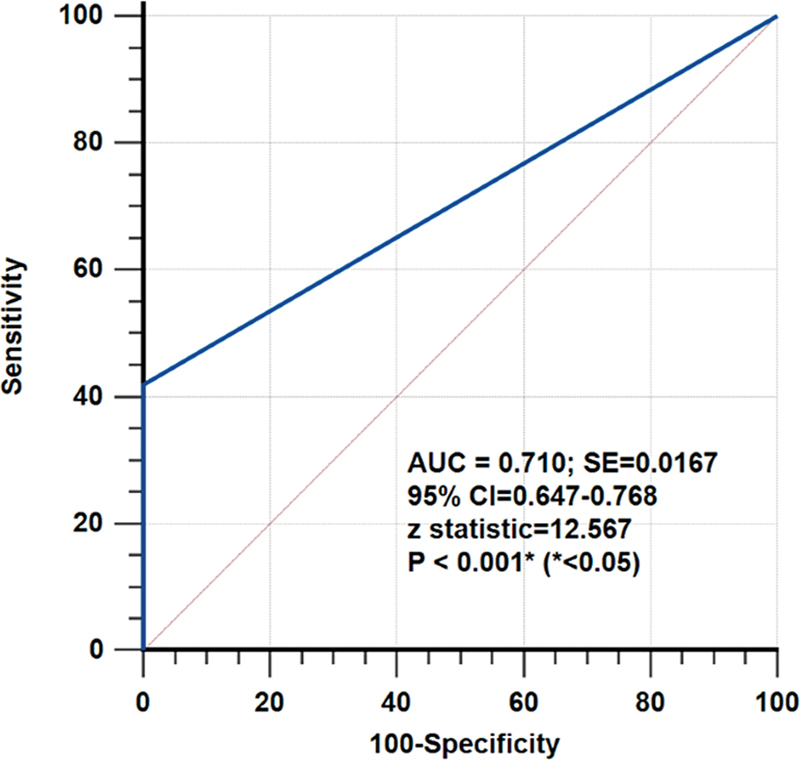
Receiver operating characteristics analysis of ^18^ FDG PET/CT for the suggested primary site in patients with carcinoma of unknown primary (considering unidentified primary sites as false negative). AUC, area under the curve; CI, confidence interval; CT, computed tomography; ^18^ FDG, 18F-fluorodeoxyglucose; PET. positron emission tomography; SE, standard error.

During the study period (August 2017–December 2023), 243 patients with CUP were referred for ^18^ FDG PET/CT. Thirteen patients (13/243) with indeterminate biopsy were excluded; 230 patients with CUP and definitive histopathological findings were recruited. In the study cohort of 230 patients, morphological imaging (such as CT, MRI, and ultrasound) and endoscopic procedures (in few patients) were inconclusive for primary tumor focus. Therefore, they were referred for ^18^ FDG PET/CT imaging. The mean age of the cohort was 58 ± 14 years (63% male and 37% female) having a mean body mass index of 26.818 ± 5.433 kg/m ^2^ and mean FBS 111 mg% (range: 74–199 mg%) ( Table 1 ). In 138 (60%) patients, ^18^ FDG PET/CTs were found to have a hypermetabolic focus (mean size: 43 mm ± 37; mean SUVmax: 10 ± 5.8) suggestive of primary tumor and subjected to biopsy ( Table 1 ). There was no statistically significant difference in SUVmax of suspected primary tumor and metastases ( *p* < 0.05). Out of 138 patients, biopsy of hypermetabolic foci was positive for primary tumor in 127 (92%) giving a detection rate of 55% (127/230) ( Fig. 1 ). In the remaining 11 patients, the biopsy of hypermetabolic foci turned out to be benign (11/138, 8%) ( Table 1 ). Distribution of hypermetabolic foci and biopsy findings are mentioned in Table 2 (pleuropulmonary being the most common site with the highest biopsy yield). Sensitivity and PPV of ^18^ FDG PET/CT were 58 and 92%, respectively. As remaining 92/230 patients with a negative ^18^ FDG PET/CT for suspected suggestive primary tumor did not undergo biopsy, TN results and specificity could not be calculated (40% FN) ( Table 3 ; Fig. 2 ). Comparing patients having positive ^18^ FDG PET/CT and TP, biopsy of hypermetabolic lesions (TP: 127/230) and a negative PET/CT (FN: 92/230) did not show any difference between demographic and tumor histology ( Table 4 ). ROC curve revealed good diagnostic strength of ^18^ FDG PET/CT for detecting unknown primary (AUC 0.710; *p* ≤ 0.001; standard error: 0.0167; confidence interval: 0.647–0.768) ( Fig. 3 ).

**Table 1:** Study demographics of baseline ^18^ FDG PET/CT in patients with CUP

Variables	*N* = 230
Age (y)	
Mean ± SD	58 ± 14
Median (range)	60 (59–89)
Gender (male: female)	144:86 (63:37%)
BMI (kg/m ^2^ ), mean ± SD	26.818 ± 5.433
Obesity (≥27.5 kg/m ^2^ )	85 (36%)
Suggested primary based on ^18^ FDG PET/CT	138 (60%)
Biopsy outcome on suggested primary ( *n* = 138)	
Positive:negative, *n* (%)	127:11 (92:8%)
^18^ FDG PET/CT-based biopsy-proven primary in CUP ( *n* = 230)	127 (55%)
Size of ^18^ FDG PET/CT-based suggested primary tumor (mm)	
Mean ± SD	43 ± 37
Median (range)	34 (10–199)
SUVmax	
Mean ± SD	10.0 ± 5.8
Median (range)	9.0 (2.5–31.0)

Abbreviations: BMI, body mass index; CT, computed tomography; CUP, carcinoma of unknown primary; ^18^ FDG, 18F-fluorodeoxyglucose; PET, positron emission tomography; SD, standard deviation; SUV, standardized uptake value.

**Table 2:** Comparison of the suggested primary site on ^18^ FDG PET/CT with biopsy outcome in patients with CUP

Suggestive primary on PET/CT	Total (138)	Biopsy positive (TP = 127)	Biopsy negative (FP = 11)	*t* -test	*p* -Value
Stomach	10 (7%)	8 (6%)	2 (18%)	2.236	0.135
Esophagus	3 (2%)	3 (2%)	0 (0%)	0.223	0.637
Colon	11 (8%)	10 (8%)	1 (9%)	0.014	0.907
Hepatopancreatobiliary	12 (9%)	10 (8%)	2 (18%)	1.253	0.263
Head and neck CA	15 (11%)	15 (12%)	0 (0%)	1.473	0.225
LPD	15 (11%)	13 (10%)	2 (18%)	0.677	0.411
Pleuropulmonary CA	48 (35%)	46 (36%)	2 (18%)	1.736	0.188
Breast	3 (2%)	3 (2%)	0 (0%)	0.223	0.637
Musculoskeletal	9 (7%)	8 (6%)	1 (9%)	0.155	0.694
Kidney–bladder	7 (5%)	7 (6%)	0 (0%)	0.694	0.405
Male genital (2 prostate)	2 (1%)	1 (1%)	1 (9%)	3.993	0.046 a
Female genital (2 ovarian; 1 endometrial)	3 (2%)	3 (2%)	0 (0%)	0.223	0.637

Abbreviations: CA, carcinoma; CT, computed tomography; CUP, carcinoma of unknown primary; ^18^ FDG, 18F-fluorodeoxyglucose; FP, false positive; LPD, lymphoproliferative disorder; PET, positron emission tomography; TP, true positive.

a *p* < 0.05.

**Table 3:** Contingency table of ^18^ FDG PET/CT-based suggested primary site in patients with CUP

^18^ FDG PET/CT findings on CUP	Positive biopsy on suggested primary	Negative biopsy on suggested primary	
Positive for suggestive primary	127 (TP)	11 (FP)	138 (all positive)
Biopsy not performed			
Negative for suggestive primary	92 (FN)		92 (all negative)
			230 (total)
Sensitivity = 58% PPV = 92%			

Abbreviations: CT, computed tomography; CUP, carcinoma of unknown primary; ^18^ FDG, 18F-fluorodeoxyglucose; FN, false negative; FP, false positive; PET, positron emission tomography; PPV, positive predictive value; TP, true positive.

Note: Detection rate: 55%; FP rate: 5%; sensitivity: 58%; and PPV: 92%.

**Table 4:** Comparison of TP and FN cohorts based on ^18^ FDG PET/CT findings in patients with CUP

Variables	TP ( *n* = 127)	FN ( *n* = 92)	*t* -test	*p* -Value
Age (y), mean ± SD	58 ± 12	58 ± 14	0.000	1.000
Male:female, *n* (%)	91:47 (66:34%)	53:39 (58:42%)	1.506	0.219
BMI, mean ± SD	26.736 ± 5.313	27.102 ± 5.762	0.486	0.628
Obesity (≥27.5 kg/m ^2^ ), *n* (%)	43 (34%)	38 (41%)	1.117	0.291
Poorly differentiated adenocarcinoma, *n* (%)	8 (6%)	5 (5%)	0.101	0.751
Well to moderately differentiated adenocarcinoma, *n* (%)	68 (54%)	55 (60%)	0.778	0.378
SCC, *n* (%)	12 (9%)	9 (10%)	0.062	0.803
NE differentiation, *n* (%)	3 (2%)	4 (4%)	0.770	0.380
Others, *n* (%)	36 (28%)	19 (21%)	1.386	0.239

Abbreviations: BMI, body mass index; CT, computed tomography; CUP, carcinoma of unknown primary; ^18^ FDG, 18F-fluorodeoxyglucose; FP, false positive; NE, neuroendocrine; PET, positron emission tomography; SCC, squamous cell carcinoma; SD, standard deviation; TP, true positive.

Note: Demographic comparison of ^18^ FDG PET/CT-based TP and FN cases of primary site in patients with CUP.

*p* < 0.05.

## Discussion

Detection of primary tumor site in CUP has been a challenge for oncologist as it impacts the survival of patients. 11 CUP is considered a diagnostic dilemma for imaging fraternity as the primary tumor cannot be detected even at autopsy in almost two-thirds of patients. 11 In 2008, a multidisciplinary expert panel of oncologists, radiologists, and nuclear physicians recommended the use of ^18^ FDG PET in the diagnostic paradigm of primary tumor detection in patients with CUP. 12 But the reported detection rate of primary tumor by ^18^ FDG PET/CT in CUP is within range of 10 to 75%. 13 In this study, which is a continuation of our previously published work, 9 ^18^ FDG PET/CT has successfully identified primary tumor with a sensitivity of 58% having a fair diagnostic strength as revealed by ROC. This sensitivity is not different from our primary study (58 vs. 57%) despite a significantly high study population (patients: 230 vs. 46). 9 Similarly, studies from various part of the world document a variable detection rate of ^18^ FDG PET/CT in CUP, ranging from 0 to 80%. 14 15 Plausible explanations for this diversified detection rate could be small sample size of many studies, use of nonstandardized imaging protocol, and use of scanners with different spatial resolutions. Our finding is in concordance with the findings of Freudenberg et al, who also reported a detection rate of 57%. 16 The sensitivity of our study is higher than a recent published study having a sensitivity of 44% (28/64 patients). 13 Similarly, a published meta-analysis of 20 studies comprising 1,942 patients revealed a pooled sensitivity of 40.93%. 17 The authors of this meta-analysis also pointed out a large heterogeneity between studies, lack of randomization, and nonstandardized diagnostic workup used in these studies. The false-positive result in our study is 5% which is associated with an established nonspecific uptake of ^18^ FDG in benign inflammatory, infective lesion, or pulmonary embolism having a reported incidence of 4% in patients with cancer. 18 Interestingly, compared with our primary study, this extension study shows a significant decline in false-positive results (previous vs, current; 17 vs. 5%), and an improved learning curve of reading physicians is the most plausible explanation in this regard. However, it is imperative to realize that false-positive ^18^ FDG PET/CT findings may result in unnecessary additional invasive diagnostic procedures having an unjustified morbidities and costs. 6 This limitation emphasizes the importance and need of more tumor-specific PET tracers in the diagnostic workup of CUP.

In the present study, the FN proportion was 40% which is comparable to 48% reported by Elboga et al. 11 While Park et al failed to find a primary tumor site on ^18^ FDG PET/CT in none of 20 patients with CUP (FN: 100%). 14 Furthermore, it must be acknowledged that despite extensive workup, diagnostic yield of imaging modality for the primary tumor is less than 20%, and 70% of cases remained undiagnosed on autopsy as well. 6 The reason for this diagnostic dilemma has still not been clarified. Common plausible explanations are (1) tumor smaller than the spatial resolution of PET/CT scanner causing no appreciation of ^18^ FDG uptake due to partial volume effect; (2) some tumors with low or no avidity for ^18^ FDG such as lobular cancer of breast, bronchoalveolar carcinoma, or well-differentiated prostate cancer; and (3) progressive or delayed ^18^ FDG uptake by tumor appreciable when target-to-background ratio is increased in delayed images. 5 Common hypotheses include spontaneous regression or immune-mediated destruction of the primary tumor or the inherent small size of the primary tumor (metastatic spread is favored above local tumor growth) beyond the spatial resolution of the scanner. 5 19

Despite having a good sample size, this study has some limitations. The major limitation of our study is heterogeneity in tumor grading; additionally, we did not acquire delayed images in patients who failed to reveal a suggestive primary tumor, as it would have resulted in unjustified delay in scheduled imaging of subsequent patients. Another potential limitation is FBS <200 mg/dL as per EANM guidelines 10 adopted in this study. It is quite possible that patients with FBS between 120 and 199 mg/dL could have more FN results than those <120 mg/dL. 20

## Conclusion

We conclude that ^18^ FDG PET/CT is an effective tool for detecting primary tumor in patients with CUP, and its upfront use could preclude use of many futile diagnostic procedures. Furthermore, tumor-specific PET tracer, higher resolution scanners, and acquiring delayed images in patients with negative ^18^ FDG PET/CT study could reduce FN results in patients with CUP.
